# Suicide in Newfoundland and Labrador, Canada: a time trend analysis from 1981 to 2018

**DOI:** 10.1186/s12889-021-11293-8

**Published:** 2021-07-02

**Authors:** Nathaniel J. Pollock, Li Liu, Margo M. Wilson, Charlene Reccord, Nicole D. Power, Shree Mulay, Yordan Karaivanov, Lil Tonmyr

**Affiliations:** 1 School of Arctic and Subarctic Studies, Labrador Institute, Memorial University, Happy Valley-Goose Bay, Newfoundland and Labrador Canada; 2 Division of Community Health and Humanities, Faculty of Medicine, Memorial University, St. John’s, Newfoundland and Labrador Canada; 3 Discipline of Emergency Medicine, Faculty of Medicine, Memorial University, St. John’s, Newfoundland and Labrador Canada; 4 Health Promotion and Chronic Disease Prevention Branch, Public Health Agency of Canada, Ottawa, Ontario Canada; 5 Department of Research and Innovation, Eastern Health, St. John’s, Newfoundland and Labrador Canada; 6 Medical Services, Labrador Health Centre, Labrador-Grenfell Health, Happy Valley-Goose Bay, Newfoundland and Labrador Canada; 7 Northern Family Medicine, Discipline of Family Medicine, Faculty of Medicine, Memorial University, Happy Valley-Goose Bay, Newfoundland and Labrador Canada

**Keywords:** Suicide, Suicide prevention, Surveillance, Epidemiology, Public health, Newfoundland and Labrador

## Abstract

**Background:**

The suicide rate in Canada decreased by 24% during the past four decades. However, rates vary between provinces and territories, and not all jurisdictions experienced the same changes. This study examined suicide rates over time in the province of Newfoundland and Labrador.

**Methods:**

We used cross-sectional surveillance data from the Canadian Vital Statistics Death Database to examine suicide rates in Newfoundland and Labrador from 1981 to 2018. We calculated annual age-standardized suicide mortality rates and used joinpoint regression to estimate the average annual percent change (AAPC) in suicide rates overall and by sex, age group, and means of suicide.

**Results:**

From 1981 to 2018, 1759 deaths by suicide were recorded among people in Newfoundland and Labrador. The age-standardized suicide mortality rate increased more than threefold over the study period, from 4.6 to 15.4 deaths per 100,000. The suicide rate was higher among males than females, and accounted for 83.1% of suicide deaths (*n* = 1462); the male-to-female ratio of suicide deaths was 4.9 to 1. The average annual percent change in suicide rates was higher among females than males (6.3% versus 2.0%). Age-specific suicide rates increased significantly for all age groups, except seniors (aged 65 or older); the largest increase was among youth aged 10 to 24 years old (AAPC 3.5; 95% CI, 1.6 to 5.5). The predominant means of suicide was hanging/strangulation/suffocation, which accounted for 43.8% of all deaths by suicide.

**Conclusions:**

The suicide rate in Newfoundland and Labrador increased steadily between 1981 and 2018, which was in contrast to the national rate decline. The disparity between the provincial and national suicide rates and the variations by sex and age underscore the need for a public health approach to prevention that accounts for geographic and demographic differences in the epidemiology of suicide.

**Supplementary Information:**

The online version contains supplementary material available at 10.1186/s12889-021-11293-8.

## Background

Suicide is a complex public health problem that requires a comprehensive and sustained prevention effort [1]. Suicide rates are influenced by a constellation of social, economic, environmental, and psychological factors in the population [2–5]. In 2017, suicide was the second leading cause of injury deaths globally and ranked 14th among the leading causes of years of life lost [6]. Since 1990, suicide rates have decreased worldwide by 32%, although rates and patterns over time differ between and within countries [7].

In Canada, the suicide rate declined by 24% between 1981 and 2017 [8]. The 2017 age-standardized suicide rate was 11.4 deaths per 100,000 people, and there were more than 4000 recorded suicide deaths on average each year between 2013 and 2019 [9]. In 2017, the majority of deaths by suicide were among males (76%) [8], and the suicide rate for males was more than three times higher than the rate for females [8]. In 2017, suicide rates were highest among adults aged 45 to 64 years [8] and suicide was the second leading cause of death among youth aged 10 to 19 years [10], though a relatively small percentage of all suicides (6.2%) occurred in this young age group [9]. Data from 2018, showed that suffocation-related suicide deaths (including hanging and strangulation) were the predominant means of suicide in Canada, accounting for 53.2% of all suicides, followed by poisoning (18%) and firearms (13.7%) [11].

At the provincial and territorial level, suicide rates vary substantially [12]. In 2018, Saskatchewan had the highest age-standardized rate among the provinces (20.2 suicide deaths per 100,000 population), and Prince Edward Island had the lowest (4.7 per 100,000) [13]. The 2018 suicide rates in the three northern territories (Yukon, Northwest Territories, and Nunavut) were higher than in any province; among the territories, the rate was highest in Nunavut (45 per 100,000) [13]. Previous studies found that compared to the national suicide rate, the largest rate disparities were in the territories and northern health regions in many of the provinces [12, 14, 15].

Historically, Newfoundland and Labrador’s suicide rate was among the lowest in Canada [12, 16, 17]. In 2012, the province’s age-standardized rate was 7.8 suicide deaths per 100,000 population, compared with the national rate of 10.4 per 100,000 [12]. Recently, families and communities have raised concerns about suicide ‘clusters’ [18], a situation in which several suicide attempts and/or deaths occur close to one another in time and place [19], and large regional differences within the province have been observed. A 2016 study found that the suicide rate in Labrador was four times higher than the rate in Newfoundland, the island portion of the province [14]. Previous research reported similar results [20] and a comparable pattern of regional disparities has been found for suicide attempts [21].

In 2016, the Government of Newfoundland and Labrador released the report titled *Towards Recovery: The Mental Health and Addictions Action Plan for Newfoundland and Labrador* [22], which presented a comprehensive approach for reforming mental health services and policies. One of the recommendations of the report was to design and implement a provincial suicide prevention strategy. This study was developed to provide a baseline assessment of the epidemiology of suicide in the province. The objective was to examine temporal trends in suicide rates in Newfoundland and Labrador overall and by sex, age group, and means of suicide.

## Methods

### Data source

We conducted this study with cross-sectional surveillance data from the Canadian Vital Statistics Death Database. Vital statistics are based on death certificates completed by physicians at the time of death; certificates include information on demographics and the cause of death. The underlying cause is coded using the World Health Organization’s *International Statistical Classification of Diseases and Related Health Problems*, Ninth Revision (ICD-9) for 1981 to 1999 and the Tenth Revision (ICD-10) for 2000 to 2018. Provincial and territorial governments maintain registries of all deaths for their jurisdictions and share data annually with Statistics Canada to create a national registry, the Canadian Vital Statistics Death Database.

We extracted aggregated data on all deaths by suicide in Newfoundland and Labrador among people aged 10 years or older for the 1981-to-2018 period based on the usual place of residence field from the national registry. Suicide deaths were identified as those with ICD-9 codes E950-E959 (from 1981 to 1999) and ICD-10 codes X60-X84 and Y87.0 (from 2000 to 2018) for intentional self-harm. Variables included sex (female, male), age group (10–24, 25–44, 45–64, 65+), and suicide means (i.e. the method, mechanism, or direct cause/source of the fatal injury), which were stratified into three groups: hanging/strangulation/suffocation (E953, X70), firearms/explosives (E955, X72-X75), and “other,” which captured poisoning (E950-E952, X60-X69) and all other means (E954, E956-E959, X71, X76-X84, Y87.0). For the population data, we used census counts and annual intercensal estimates from Statistics Canada.

### Setting

This study examined suicide in Newfoundland and Labrador, the easternmost province in Canada. In 2016, the provincial population was relatively small (519,715) and the majority of residents lived in rural population centres (23.7%) or rural communities (41.9%) [23].  Overall, the population is spread over two large geographic areas—an island (Newfoundland) and a northern mainland region (Labrador), which together had a combined landmass of 370,514 km^2^. Health services are provincially-funded and delivered by the four regional health authorities, three of which are primarily rural.

### Statistical analysis

We analyzed the suicide data with descriptive statistics (frequencies and percentages), then examined trends in suicide rates overall, and by sex, age group, and means from 1981 to 2018. To calculate the crude and age-standardized incidence rates of suicide mortality for each year, we used census and annual intercensal population data (10 years or older) for the denominator and adjusted for age with the 2011 Canadian Standard Population.

1. Age-standardized mortality rate (ASMR) per 100,000 population:

Age-adjusted with 5-year age groups

$$ ASMR=\sum \limits_{i= 1}^n\left(\frac{\sum \limits_{j= 1}^m{N}_ij}{\sum \limits_{j= 1}^m{pop}_j}\times \frac{pop{ 2011}_i}{ pop 2011}\right)\times 100,000 $$

*N*: number of suicide deaths

*n*: total number of 5-year age bands, and i = 1, 2, … …, 16, 17, represent age groups 10–14, 15–19, …, 85–89, and 90+

*m*: number of years combined

for overall and by gender: m = 1

for age group and by cause of death: m = 2

*pop*: population in a specific year

*pop2011*: total population in 2011

*pop2011*_*i*_: population in 2011 for *i*th 5-year age group

(2) Age-specific mortality rate (crude rate) per 100,000 population:

$$ \frac{\sum \limits_{j=1}^m{N}_j}{\sum \limits_{j=1}^m{pop}_j} $$

Suicide rates overall and by sex and means were age-adjusted with 5-year age groups. For age group and suicide means, we combined two years of suicide and population data (1981/1982, 1983/1984 … etc.) to eliminate small counts (< 5 deaths) and increase the stability of the rates. For age-specific suicide rates, we used four groups representing major life stages: youth (10–24), young adults (25–44), middle-age adults (45–64), and seniors (65 or older). For age group and suicide means, we calculated rates overall and rates for males. Due to small cell counts, we did not calculate age group or means-specific suicide rates for females. To calculate rates over time, poisoning deaths were also grouped with “other” means because of small annual counts.

We used the Joinpoint Regression Program Version 4.7.0.0 to perform a trend analysis on the suicide rates and to determine the average annual percent change (AAPC) and 95% confidence intervals. We used the default parameters in the software which allowed for a maximum of 5 joinpoints, and required a minimum of two observations from a joinpoint to either end of the data, and a minimum of two observations between two joinpoints. We applied the Bonferroni correction to overall significance levels in permutation tests since there were multiple tests by sex, age group, and means. Finally, we calculated incidence rate ratios for the observed and modeled standardized and age-specific rates to provide a measure of the change between the earliest and most recent year/period for each subgroup. We used the REporting of studies Conducted using Observational Routinely-collected Data (RECORD) guidelines to report the results [24].

### Ethics approval

This study aggregated a de-identified dataset available through a data sharing agreement between the Public Health Agency of Canada and Statistics Canada. In accordance with the federal government’s *Tri-Council Policy Statement: Ethical Conduct for Research Involving Humans* [25] and the policies of the Newfoundland and Labrador Health Research Ethics Authority [26], the use of this dataset did not require research ethics board approval.

## Results

From 1981 to 2018, there were 1759 deaths by suicide recorded among people aged 10 years or older in Newfoundland and Labrador. The majority of deaths by suicide were among males (83.1%) and the ratio of male-to-female deaths was 4.9 to 1. Twenty percent of suicides (20.4%; *n* = 358) were among youth aged 10 to 24 years; 35.8% (*n* = 629) and 33.3% (*n* = 586) of suicide deaths occurred among young adults (25–44 years) and middle-age adults (45–64 years); 10.6% (*n* = 186) of suicide deaths were among seniors (65 years or older).

**Fig. 1:**
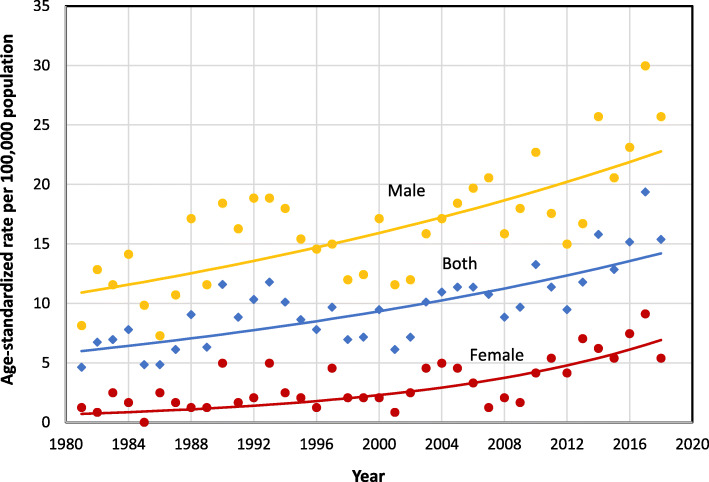
Age-standardized suicide mortality rates, by sex, Newfoundland and Labrador, 1981–2018

The annual number of suicide deaths ranged from a total of 22 in 1981 to a peak of 92 in 2017 (Additional file 1), with an average of 46.3 suicides per year. The age-standardized mortality rate (ASMR) more than tripled (incidence rate ratio 3.3; Table 1) during the study period, rising from 4.6 deaths per 100,000 people in 1981 to 15.4 in 2018 (Fig. 1; Additional file 1). The change was significant, with an average increase of 2.4% per year (95% CI: 1.7 to 3.0, < 0.001; Table 1).

**Table 1:** Average annual percent change in suicide rates in Newfoundland and Labrador, 1981–2018

	Sex	Age group	Cause of death	Start year(s)	End year(s)	AAPC	Lower CI	Upper CI	***P***-Value	Rate ratio 2018/1981 Observed	Rate ratio 2018/1981 Modeled
All	Both sexes	All	All	1981	2018	2.4*	1.7	3.0	< 0.001	3.3	2.4
Sex	Female	All	All	1981	2018	6.3*	2.3	10.5	< 0.001	4.3	9.7
	Male	All	All	1981	2018	2.0*	1.3	2.7	< 0.001	3.2	2.1
Age group^**^	Both sexes	10–24	All	1981–1982	2017–2018	3.5*	1.6	5.5	< 0.001	2.4	3.4
		25–44	All	1981–1982	2017–2018	2.9*	1.9	3.9	< 0.001	3.5	2.8
		45–64	All	1981–1982	2017–2018	1.9*	0.8	2.9	< 0.001	2.9	1.9
		65+	All	1981–1982	2017–2018	−0.2	−1.6	1.2	0.8	1.8	0.9
	Male	10–24	All	1981–1982	2017–2018	2.7*	0.9	4.6	< 0.001	1.7	2.6
		25–44	All	1981–1982	2017–2018	2.5*	1.7	3.3	< 0.001	3.4	2.4
		45–64	All	1981–1982	2017–2018	1.7*	0.6	2.9	< 0.001	2.5	1.9
		65 +	All	1981–1982	2017–2018	−0.3	−1.5	0.9	0.6	1.5	0.9
Means of suicide^**^	Both sexes	All	Firearms	1981–1982	2017–2018	−0.4	−1.7	0.9	0.5	1.2	0.9
		All	Others	1981–1982	2017–2018	2.0*	0.6	3.3	< 0.001	3.0	2.0
		All	Suffocation	1981–1982	2017–2018	4.0*	3.1	4.9	< 0.001	6.5	4.1
	Male	All	Firearms	1981–1982	2017–2018	−0.2	−1.4	1.0	0.8	1.4	0.9
		All	Others	1981–1982	2017–2018	1.6*	0.1	3.1	< 0.001	1.6	1.8
		All	Suffocation	1981–1982	2017–2018	3.5*	2.6	4.4	< 0.001	5.4	3.4

* Statistically significant. For non-stratified analyses, a *p*-value of 0.05 was used as the cut-off for statistical significance. Bonferroni corrections were applied for stratified analysis. For analyses by age group, a *p*-value of 0.01was used as the cut-off. For analyses by cause of death, a *p*-value for 0.015 was used

** Due to small cell counts, rates were calculated by combining annual deaths into two-year periods

*AAPC* average annual percent change, *CI* confidence interval

The ASMR among females rose from 1.2 suicide deaths per 100,000 in 1981 to 5.4 in 2018 (Fig. 1; Additional file 1). The ASMR among males rose from 8.1 suicide deaths per 100,000 in 1981 to 25.7 in 2018. The average annual percent increase was three times higher among females than males (6.3% versus 2.0%; Table 1), although annual ASMRs were 2.9 to 16.6 times higher among males (Fig. 1; Additional file 1).

**Fig. 2:**
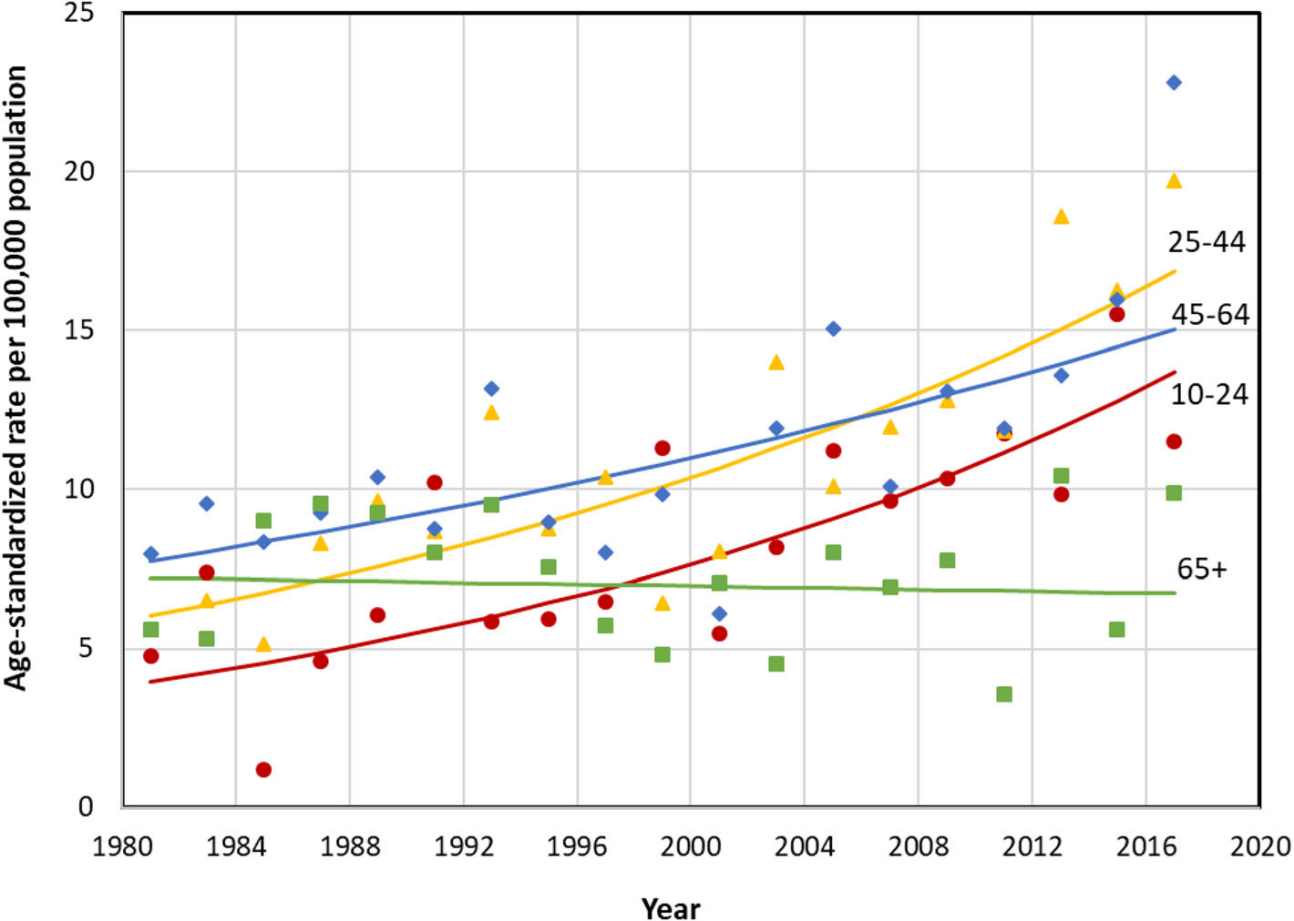
Age-standardized suicide mortality rates, by age group, Newfoundland and Labrador, 1981–2018 (based on combined deaths for every 2 years) (see attached)

Age-specific suicide rates increased significantly for all age groups, except seniors (Table 1, Fig. 2). The largest suicide rate increase was among youth aged 10 to 24 (AAPC 3.5; 95% CI, 1.6 to 5.5; Table 1). In 2017–2018, the rate for both sexes combined was highest among 45- to 64-year olds (22.8 per 100,000) and lowest among seniors aged 65 years or older (9.9 per 100,000). In 1981–1982, the ASMR had been highest at ages 45 to 64, and lowest at ages 10 to 24 (Fig. 2). The suicide rate declined slightly for seniors, but the change was not significant. In all other age groups, the rate changes over time for both sexes paralleled the pattern among males.

**Fig. 3:**
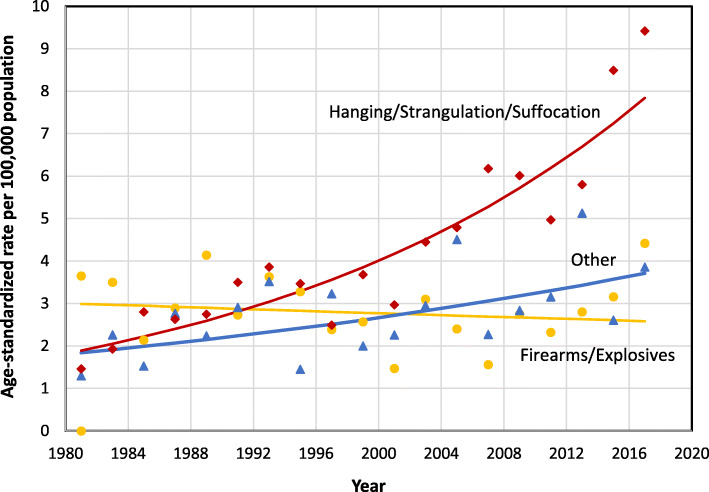
Age-standardized suicide mortality rates, by means of suicide, Newfoundland and Labrador, 1981–2018. *Notes:* Based on combined deaths for every 2 years; ICD-9 and ICD-10 codes for suicide by cause (means) include Hanging, Strangulation, or Suffocation (E953, X70), Firearms or Explosives (E955, X72-X75), and all other means (E950-E952, E954, and E956-E959; X60-X69, X71, X76-X84, and Y87.0)

Hanging/strangulation/suffocation was the predominant means of suicide, accounting for 43.8% of all suicide deaths for both sexes combined and 45.3% for males. Firearms accounted for one-third of suicide deaths among males (Table 2). Among females, poisoning was the leading means, accounting for 40.4% of all suicide deaths. The ASMR for suicide by hanging/strangulation/suffocation rose significantly between 1981 and 2018 (AAPC = 4.0; 95% CI, 3.1 to 4.9) from 1.5 to 9.4 per 100,000 population (Table 1, Fig. 3).

**Table 2:** Number and percentage of suicide deaths, by means and sex, Newfoundland and Labrador, 1981–2018

Means of suicide (ICD-9, ICD-10)	Both sexes		Male		Female	
	Deaths, n=	%	Deaths, n=	%	Deaths, n=	%
Hanging/Strangulation/ Suffocation (E953, X70)	771	43.8	663	45.3	108	36.4
Firearms/Explosives (E955, X72-X75)	521	29.6	493	33.7	28	9.4
Poisoning^a^ (E950-E952, X60-X69)	288	16.4	168	11.5	120	40.4
Other (E954, E956-E959, X71, X76-X84, Y87.0)	179	10.2	138	9.4	41	13.8
Total	1759	100.0	1462	100.0	297	100.0

^a^ Suicide deaths due to poisoning are reported separately from “Other” in this table because it includes a pooled count; due to small numbers, annual counts or rates are not reported for poisoning in the manuscript. Estimates of time trends by suicide means include poisoning in the “Other” category

The suicide rate among people who died by firearms/explosives decreased slightly over time, but the trend was not significant. In 1981, firearms/explosives were the leading cause of fatal injuries in suicide deaths. This changed in the early 1990s, when hanging/strangulation/suffocation became the leading cause (Fig. 3). Trends over time by suicide means were similar for males and both sexes combined.

## Discussion

In contrast to declines in suicide rates in Canada [8], the suicide rate in Newfoundland and Labrador increased significantly between 1981 and 2018. In 2018, the provincial suicide rate was also 1.35 times higher than the national rate (15.4 versus 11.4 suicide deaths per 100,000 pop.) [8]. We found that the majority (83.1%) of suicide deaths were among males, who also had the highest suicide rates across age groups; this is similar to the pattern nationally [8, 12]. The average annual suicide rate increase was higher among females (6.3%), which is a trend that mirrored rate increases among adolescent, young adult, and middle-aged females in Canada [8], and among females in other high-income nations [27–29]. Suicide mortality rose in all age groups, except for seniors, among whom rates were stable over time. In 2018, age-specific suicide rates were highest among 25- to 44-year-olds.

Between 1981 and 2018, the rate of suicide by hanging/strangulation/suffocation increased for both sexes; among males it was the leading means of suicide (45.3%), followed by firearms (33.7%). The predominant means of suicide among females was poisoning (40.4%). We found that rates of suicide by firearms/explosives were relatively stable over time in Newfoundland and Labrador, which contrasted with the 3.3% (95% CI: − 3.8 to − 2.8) decrease in the rate of firearm-related suicide nationally during the same period [11].

### Long-term trends in suicide mortality

After World War II, the suicide rate in Canada doubled from 7.4 suicide deaths per 100,000 in 1951 and to 14.8 in 1983 [30]. However, in the early 1980s, the national rate began to drop [8, 30], likely driven by declines in the largest provinces (Quebec, Ontario, and British Columbia) [13], particularly since 2000. In Newfoundland and Labrador, suicide rates were comparatively low during the same period [17, 31, 32]. Previous studies found that between 1951 and 1979, annual crude rates in the province ranged from 1.2 to 4.8 deaths per 100,000 [17, 31, 32]. In contrast to national declines over the past four decades [8], we found that between 1981 and 2018, the suicide rate in Newfoundland and Labrador tripled, from 4.6 to 15.4 suicide deaths per 100,000. During the latter half of this period, two other provinces with small and relatively rural populations [23] also experienced suicide rate increases of more than 50%: the rate in Saskatchewan rose from 12.4 to 20.2 suicide deaths per 100,000, and the rate in in Nova Scotia increased from 8.2 to 12.5 [13].

International evidence has shown that changing social, economic, and environmental conditions can impact suicide rates [2–5, 33]. However, the effects vary over time and across geographic contexts, and may be mitigated through economic and social policy interventions. For example, the “crisis effect” of the 2008 global recession was not universal. Suicide rates increased in European countries where unemployment rose precipitously, such as in Greece, the Netherlands, and England [33, 34]. By contrast, nations with better income support programs did not experience increased rates of suicide [33, 34].

In the United States, suicide rates had been rising prior to the 2008 recession, and the upward trend continued thereafter [36]; this suggests that a broader constellation of determinants may be at play. Our results showed a similar pattern in Newfoundland and Labrador—there was no year or period in which the trend in suicide mortality diverged from the steady increase. The provincial upturn in suicide rates since 1981 preceded not only the global economic crisis that occurred in the latter part of the study period, but also a regional crisis that occurred in the decade prior.

### Socio-economic changes and suicide rates in Newfoundland and Labrador

Historically, the fishing industry has been a major economic driver in Newfoundland and Labrador [37]. In 1992, a federal moratorium on the Atlantic cod fishery resulted in the largest industrial layoff in Canadian history as an estimated 40,000 people who worked in the sector lost their jobs [37, 38]. The moratorium magnified the province’s already high rates of unemployment and ushered in a period of social and economic change [37–39].

The overwhelming sense of loss from the end of the fishery also negatively affected mental health, especially for families who were reliant on the industry for their livelihood [38–40]. Despite the often observed link between economic crises and suicide [3, 36], our results did not show a significant change in suicide rates immediately following the moratorium, though there appeared to be a slight non-significant increase in the observed rates and number of deaths. Instead, the moratorium and its fallout may have had latent effects on suicide rates, with risks accumulating over the long term, as has been found with other disasters [41].

Over the past four decades, rural communities in Newfoundland and Labrador have experienced considerable financial hardship and a loss of cultural identity and social connectedness [38, 39, 42]. After the moratorium, many young people and families left rural communities for education, retraining, and work in urban areas of the province and elsewhere in Canada [38, 39, 42]. This outmigration led to a 12% population decline (71,504 people) between 1992 and 2007, which disproportionately affected rural communities. Consequently, many services, including health and mental health care, have become increasingly concentrated in regional and urban centres, which has further entrenched inequities for rural people.

Cumulatively, the social and economic changes experienced by rural communities in Newfoundland and Labrador may have impacted suicide rates and rate disparities. However, we did not disaggregate data by rural-urban status, and so were not able to assess geographic differences in mortality within the province. Cross-sectional data from 2009-2011 showed that the suicide rate was nearly two times higher in the most rural areas of the province compared to urban areas [43]. A study with provincial medical examiner data also found that compared to urban decedents, rural people who died by suicide were younger, more likely to be male, and more likely to have died by hanging [44]. Given that the rural population accounts for more than 40% of the provincial population [23], larger rate increases in rural communities and widening rural-urban disparities in suicide may have impacted the overall suicide rate increase, along with rate increases among youth, males, and for suicide by hanging.

Prior research found that suicide rates were higher in Labrador than in Newfoundland [14, 20], and up to twenty times higher in Inuit and Innu First Nation communities in particular [14]. The disparity was largest for females in Nunatsiavut, the Inuit region in northern Labrador, who had an age-standardized suicide mortality rate that was 31.5 times (95% CI 18.3, 54.4) higher than the rate among females in Newfoundland (ASMR 75.5 versus 2.4 per 100,000) [14]. The presence of such disparities warrants targeted interventions as a part of a comprehensive suicide prevention strategy. Evidence of localized variance should also help direct future research about the effect that inequities have on suicide rates among females and in the province overall.

It is possible that socio-economic changes and widening geographic disparities have contributed to the increased suicide rates in the province. However, given the complex and interconnected nature of population and individual-level risks for suicide, it is difficult to pinpoint how and which specific factors might have contributed to the rate increases. As a surveillance study, this was not something we were able to achieve, though it does warrant further investigation.

### Limitations

The results of this study should be considered in the context of several limitations. Mortality data were coded with two different versions of the *International Statistical Classification of Diseases and Related Health Problems*: ICD-9 codes E950–959 were used to identify suicide deaths for the 1981-to-1999 period and ICD-10 codes X60-X84 and Y87.0 were used for the 2000-to-2018 period. Although changes in how deaths were coded could have influenced the results, comparability studies have shown that differences between versions of the ICD have limited-to-negligible impacts on patterns of suicide mortality [45–47].

Another consideration is that suicide deaths are sometimes misclassified as unintentional or undetermined injuries [20, 31, 48]. An analysis of suicide data for the 1997-to-2001 period found that vital statistics data in Newfoundland and Labrador accurately captured only 83% of suicide deaths compared with data from the provincial medical examiner [20]. Misclassification and underreporting of suicide may be related to factors including a lack of resources for death investigations, particularly in rural areas [31], as well as to social-cultural beliefs about suicide.

The accuracy of suicide data for Newfoundland and Labrador likely improved during our study period owing to standardization of death investigations after the appointment of a chief forensic pathologist in 1986 [32] and passage of the *Fatalities Investigation Act* in 1995. Evidence from other provinces has shown relatively high rates of concordance between vital statistics and medico-legal investigation data on suicide [49]. Notwithstanding this, minor discrepancies were observed between the number of suicide deaths in our data, which were extracted by the Public Health Agency of Canada, and publicly available data reported by Statistics Canada. For the most recent period of public data (2000-to-2018), there were 12 additional suicide deaths in the Statistics Canada data, an average of less than 1 death per year [50]. The influence of this difference on suicide rates is likely marginal.

Another limitation was that suicide rate estimates for some subgroups were based on a small number of deaths. This is a common challenge for research on suicide [15], particularly in regions with small populations. Stratified analyses of small-count data are especially problematic because they can yield unstable rates with wide confidence intervals—a minor change in the number of deaths in a single year can effect rate estimates and convey an artificial sense of variation over time.

To minimize the potential effects of rate instability and to reduce the risk of identifiability related to small numbers, we made several analytical choices. For temporal trends by age group and suicide means, we pooled data into two-year groups and calculated rates for both sexes combined and for males, but not females. For annual rates by method, we grouped deaths by poisoning with “other” suicide means including drowning, jumping, and laceration/cutting. For age-specific suicide rates, we used age ranges that represented broad life course stages. A possible consequence of this approach was that it may have obscured epidemiological patterns and risks in specific developmental periods such as adolescence (15–19 years old) or later life (80 years or older).

### Advancing a public health approach to suicide prevention

In contrast to global and national declines in suicide mortality over the past three decades [7, 8], the suicide rate in Newfoundland and Labrador tripled between 1981 and 2018. This evidence underscores the need for a public health approach to suicide prevention. Research from high-income countries has demonstrated that universal strategies such as mental health policies, means restriction, and community-level interventions have been effective at preventing suicide [51–54]. Similarly, observational studies have found that policies related to unemployment protections and minimum wage increases can help buffer against increased suicide rates, especially during periods of economic insecurity [35, 55–57].

In the Canadian context, the provincial government in Quebec created a suicide prevention strategy in 1998. It included interventions such as improving the availability and quality of mental health services, providing timely follow-up care, efforts to de-stigmatize help-seeking behaviour, and reducing access to lethal means [58]. From 2000 to 2018, the suicide rate in Quebec fell by nearly half (− 44.5%), from 17.3 to 9.6 suicide deaths per 100,000 people [13]. This apparent success may provide useful direction for other provinces.

In 2017, the provincial government in Newfoundland and Labrador committed to develop a suicide prevention action plan as part of a broader effort to improve mental health services and outcomes [22]. Given the results of our study, especially where they contrast with the epidemiology of suicide nationally [8], interventions in the provincial plan should be tailored to address the demographic and geographic patterns of suicide that have emerged in the province. Further understanding sex differences in suicide, and their relationship to social and economic changes, may help align universal interventions and public policies with sex-specific suicide risks. The rapid suicide rate increases among children and youth and younger adults may be a signal that young people and families are facing unique stresses that require targeted programs and supports. Along with sex- and age-specific considerations, suicide prevention needs to address place-based differences in service delivery and outcomes. This requires a focus on the subgroups that experience the largest disparities, including Indigenous communities in Labrador [14].

Amidst an ongoing social and economic transition, a public health approach to prevention may help decrease the number of people that die by suicide in Newfoundland and Labrador. To achieve this will require prioritizing suicide prevention in public policy, setting measurable goals for reducing the incidence of suicide attempts and deaths, and evaluating the impact of interventions as a way to track progress towards lower suicide rates [1, 15, 54, 59].

## Conclusion

This study examined patterns of suicide mortality in Newfoundland and Labrador over a period of nearly four decades. While suicide rates decreased in Canada, rates in Newfoundland and Labrador rose among both sexes and most age groups between 1981 and 2018. During this time, the province experienced significant social and economic changes. Against this backdrop, important demographic and health changes have occurred, although the specific reasons for the steady increase in suicide rates remain unclear.

Our findings underscore the need to accelerate efforts to understand the factors that have driven the suicide rate increases in Newfoundland and Labrador, and to develop evidence-based policy, community, and clinical interventions to prevent suicide. Our study provides a baseline assessment from which to track progress and evaluate the impact of prevention initiatives. In doing so, our results reinforce the value of localized public health monitoring.

## Supplementary Information

**Additional file 1.** Number, crude, and age-standardized suicide rates by sex in Newfoundland and Labrador, 1981–2018 (See attached).

## Data Availability

Data were obtained through a data sharing agreement between the Public Health Agency of Canada and Statistics Canada. All data generated or analysed during this study are included in this article and the supplementary information files.

## Abbreviations

ICD: International Statistical Classification of Diseases and Related Health Problems
ASMR: Age-standardized mortality rate
AAPC: Average annual percent change
