# Supplementary material for: Suicide in Newfoundland and Labrador, Canada: a time trend analysis from 1981 to 2018
Source: BMC Public Health. 2021 Jul 2;21:1291. doi: 10.1186/s12889-021-11293-8 (PMC8252247; doi:10.1186/s12889-021-11293-8)
Supplement: Supplementary file 1 — Additional file 1. Number, crude, and age-standardized suicide rates by sex in Newfoundland and Labrador, 1981–2018 (See attached). [file 12889_2021_11293_MOESM1_ESM.docx]

| **Additional File 1**: Number, crude, and age-standardized suicide rates by sex in Newfoundland and Labrador, 1981-2018 | | | | | |
| --- | --- | --- | --- | --- | --- |
| **Sex** | **Year** | **Deaths by suicide** | **Population** | **Crude Rate per 100,000 Population** | **Age Standardized Rate per 100,000 Population** |
| Both sexes | 1981 | 22 | 468916 | 4.7 | 4.6 |
| Both sexes | 1982 | 32 | 471042 | 6.8 | 6.7 |
| Both sexes | 1983 | 33 | 478747 | 6.9 | 6.9 |
| Both sexes | 1984 | 37 | 482337 | 7.7 | 7.8 |
| Both sexes | 1985 | 23 | 484564 | 4.7 | 4.8 |
| Both sexes | 1986 | 23 | 484581 | 4.7 | 4.8 |
| Both sexes | 1987 | 29 | 486432 | 6.0 | 6.1 |
| Both sexes | 1988 | 43 | 488552 | 8.8 | 9.1 |
| Both sexes | 1989 | 30 | 492177 | 6.1 | 6.3 |
| Both sexes | 1990 | 55 | 494607 | 11.1 | 11.6 |
| Both sexes | 1991 | 42 | 498768 | 8.4 | 8.8 |
| Both sexes | 1992 | 49 | 501167 | 9.8 | 10.3 |
| Both sexes | 1993 | 56 | 503246 | 11.1 | 11.8 |
| Both sexes | 1994 | 48 | 500640 | 9.6 | 10.1 |
| Both sexes | 1995 | 41 | 496649 | 8.3 | 8.6 |
| Both sexes | 1996 | 37 | 492144 | 7.5 | 7.8 |
| Both sexes | 1997 | 46 | 486340 | 9.5 | 9.7 |
| Both sexes | 1998 | 33 | 478219 | 6.9 | 6.9 |
| Both sexes | 1999 | 34 | 474071 | 7.2 | 7.2 |
| Both sexes | 2000 | 45 | 471157 | 9.6 | 9.5 |
| Both sexes | 2001 | 29 | 467534 | 6.2 | 6.1 |
| Both sexes | 2002 | 34 | 466525 | 7.3 | 7.2 |
| Both sexes | 2003 | 48 | 466646 | 10.3 | 10.1 |
| Both sexes | 2004 | 52 | 466547 | 11.1 | 10.9 |
| Both sexes | 2005 | 54 | 464470 | 11.6 | 11.4 |
| Both sexes | 2006 | 54 | 461778 | 11.7 | 11.4 |
| Both sexes | 2007 | 51 | 460627 | 11.1 | 10.7 |
| Both sexes | 2008 | 42 | 462869 | 9.1 | 8.8 |
| Both sexes | 2009 | 46 | 467326 | 9.8 | 9.7 |
| Both sexes | 2010 | 63 | 472283 | 13.3 | 13.3 |
| Both sexes | 2011 | 54 | 475010 | 11.4 | 11.4 |
| Both sexes | 2012 | 45 | 476619 | 9.4 | 9.5 |
| Both sexes | 2013 | 56 | 477723 | 11.7 | 11.8 |
| Both sexes | 2014 | 75 | 478911 | 15.7 | 15.8 |
| Both sexes | 2015 | 61 | 479072 | 12.7 | 12.8 |
| Both sexes | 2016 | 72 | 480595 | 15.0 | 15.2 |
| Both sexes | 2017 | 92 | 480217 | 19.2 | 19.4 |
| Both sexes | 2018 | 73 | 478777 | 15.2 | 15.4 |
| Female | 1981 | - | 233211 | 1.3 | 1.2 |
| Female | 1982 | - | 234789 | 0.9 | 0.8 |
| Female | 1983 | 6 | 238401 | 2.5 | 2.5 |
| Female | 1984 | - | 240429 | 1.7 | 1.7 |
| Female | 1985 | - | 241749 | - | - |
| Female | 1986 | 6 | 242248 | 2.5 | 2.5 |
| Female | 1987 | - | 243292 | 1.6 | 1.7 |
| Female | 1988 | - | 244314 | 1.2 | 1.2 |
| Female | 1989 | - | 245833 | 1.2 | 1.2 |
| Female | 1990 | 12 | 246979 | 4.9 | 5.0 |
| Female | 1991 | - | 249076 | 1.6 | 1.7 |
| Female | 1992 | 5 | 250455 | 2.0 | 2.1 |
| Female | 1993 | 12 | 251792 | 4.8 | 5.0 |
| Female | 1994 | 6 | 250896 | 2.4 | 2.5 |
| Female | 1995 | 5 | 249800 | 2.0 | 2.1 |
| Female | 1996 | - | 248110 | - | - |
| Female | 1997 | 11 | 245639 | 4.5 | 4.6 |
| Female | 1998 | 5 | 242580 | 2.1 | 2.1 |
| Female | 1999 | 5 | 240781 | 2.1 | 2.1 |
| Female | 2000 | 5 | 239582 | 2.1 | 2.1 |
| Female | 2001 | - | 238342 | - | - |
| Female | 2002 | 6 | 237807 | 2.5 | 2.5 |
| Female | 2003 | 11 | 237666 | 4.6 | 4.6 |
| Female | 2004 | 12 | 237470 | 5.1 | 5.0 |
| Female | 2005 | 11 | 236353 | 4.7 | 4.6 |
| Female | 2006 | 8 | 235277 | 3.4 | 3.3 |
| Female | 2007 | - | 235089 | - | - |
| Female | 2008 | 5 | 236106 | 2.1 | 2.1 |
| Female | 2009 | - | 238239 | - | - |
| Female | 2010 | 10 | 240360 | 4.2 | 4.1 |
| Female | 2011 | 13 | 241557 | 5.4 | 5.4 |
| Female | 2012 | 10 | 242226 | 4.1 | 4.1 |
| Female | 2013 | 17 | 242654 | 7.0 | 7.0 |
| Female | 2014 | 15 | 242896 | 6.2 | 6.2 |
| Female | 2015 | 13 | 242722 | 5.4 | 5.4 |
| Female | 2016 | 18 | 243264 | 7.4 | 7.5 |
| Female | 2017 | 22 | 243182 | 9.0 | 9.1 |
| Female | 2018 | 13 | 242787 | 5.4 | 5.4 |
| Male | 1981 | 19 | 235705 | 8.1 | 8.1 |
| Male | 1982 | 30 | 236253 | 12.7 | 12.9 |
| Male | 1983 | 27 | 240346 | 11.2 | 11.6 |
| Male | 1984 | 33 | 241908 | 13.6 | 14.1 |
| Male | 1985 | 23 | 242815 | 9.5 | 9.9 |
| Male | 1986 | 17 | 242333 | 7.0 | 7.3 |
| Male | 1987 | 25 | 243140 | 10.3 | 10.7 |
| Male | 1988 | 40 | 244238 | 16.4 | 17.1 |
| Male | 1989 | 27 | 246344 | 11.0 | 11.6 |
| Male | 1990 | 43 | 247628 | 17.4 | 18.4 |
| Male | 1991 | 38 | 249692 | 15.2 | 16.3 |
| Male | 1992 | 44 | 250712 | 17.6 | 18.8 |
| Male | 1993 | 44 | 251454 | 17.5 | 18.8 |
| Male | 1994 | 42 | 249744 | 16.8 | 18.0 |
| Male | 1995 | 36 | 246849 | 14.6 | 15.4 |
| Male | 1996 | 34 | 244034 | 13.9 | 14.6 |
| Male | 1997 | 35 | 240701 | 14.5 | 15.0 |
| Male | 1998 | 28 | 235639 | 11.9 | 12.0 |
| Male | 1999 | 29 | 233290 | 12.4 | 12.4 |
| Male | 2000 | 40 | 231575 | 17.3 | 17.1 |
| Male | 2001 | 27 | 229192 | 11.8 | 11.6 |
| Male | 2002 | 28 | 228718 | 12.2 | 12.0 |
| Male | 2003 | 37 | 228980 | 16.2 | 15.8 |
| Male | 2004 | 40 | 229077 | 17.5 | 17.1 |
| Male | 2005 | 43 | 228117 | 18.8 | 18.4 |
| Male | 2006 | 46 | 226501 | 20.3 | 19.7 |
| Male | 2007 | 48 | 225538 | 21.3 | 20.6 |
| Male | 2008 | 37 | 226763 | 16.3 | 15.8 |
| Male | 2009 | 42 | 229087 | 18.3 | 18.0 |
| Male | 2010 | 53 | 231923 | 22.9 | 22.7 |
| Male | 2011 | 41 | 233453 | 17.6 | 17.6 |
| Male | 2012 | 35 | 234393 | 14.9 | 15.0 |
| Male | 2013 | 39 | 235069 | 16.6 | 16.7 |
| Male | 2014 | 60 | 236015 | 25.4 | 25.7 |
| Male | 2015 | 48 | 236350 | 20.3 | 20.6 |
| Male | 2016 | 54 | 237331 | 22.8 | 23.1 |
| Male | 2017 | 70 | 237035 | 29.5 | 30.0 |
| Male | 2018 | 60 | 235990 | 25.4 | 25.7 |
| Notes: "-" indicates suppressed counts (n) under 5 | | | | | |
